# Central form of multiple symmetric lipomatosis: a case report

**DOI:** 10.4076/1757-1626-2-8427

**Published:** 2009-08-12

**Authors:** Ahmet Tekin, Zekai Ogetman

**Affiliations:** Department of Surgery, International Medical CentreIstiklal Cad. No: 196 33100 MersinTurkey

## Abstract

Multiple symmetric lipomatosis (also known as Madelung's disease, Launois-Bensaude syndrome or benign symmetric lipomatosis) is a rare disease, the etiology of which is unknown. Multiple, symmetric, non-encapsulated lipomatous masses on the face, neck, upper arms, and upper trunk are typical in most cases. Five out of the 200 cases reported in the literature were the distal form of the disease; the rest were proximal. We describe, for the first time, a central form of the disease, simultaneously involving the lower trunk, arms and upper legs.

## Introduction

Multiple symmetric lipomatosis (MSL; also known as Madelung’s disease, Launois-Bensaude syndrome, or benign symmetric lipomatosis) is rare. It is characterized by a diffuse or circumscribed symmetrical accumulation of adipose tissue, primarily around the neck, back, shoulders and upper trunk. The cause of MSL is unknown, but up to 90% of patients have associated alcoholism.

MSL was initially described by Sir Benjamin Brodie in 1846 [1]. In 1888, Otto Madelung reported the first series of 33 patients with lipomas associated with alcoholism. In the following year, Launois and Bansaude described 65 patients with similar features [2,3]. Since then, more than 200 patients have been reported in the literature. The vast majority of these reported cases were the proximal form of MSL. Five cases of the distal form of MSL, involving the hands, feet or knees, have been reported [4-8]. We describe, for the first time, an unusual case of the central form of MSL, simultaneously involving the lower trunk, arms and upper legs.

## Case presentation

A 26-year-old male residing on the north-east Mediterranean coast was admitted to our hospital complaining of multiple palpable masses on the surface on his body. He is Turkish citizen but his mother is Caucasians and his father is Arabian. Ten years earlier, he had noticed two symmetric masses on his forearms. Two years after onset, similar masses developed progressively on the lower trunk, arms and upper legs, and gradually increased in size. A complete blood count, chemistry panel, hepatic function panel, total cholesterol, LDL cholesterol, triglycerides (lipid profile) and endocrine test (ACTH, cortizol, GH, TSH, fT3, fT4, insulin, LH, FSH, testosterone, androstenedione, estradiol, progesterone, prolactin and DHEA-SO4) results were normal. There was no history of alcohol abuse. There was no other previous illness that required medical or surgical treatment. There was no family history of a similar disorder.

**Figure 1.**
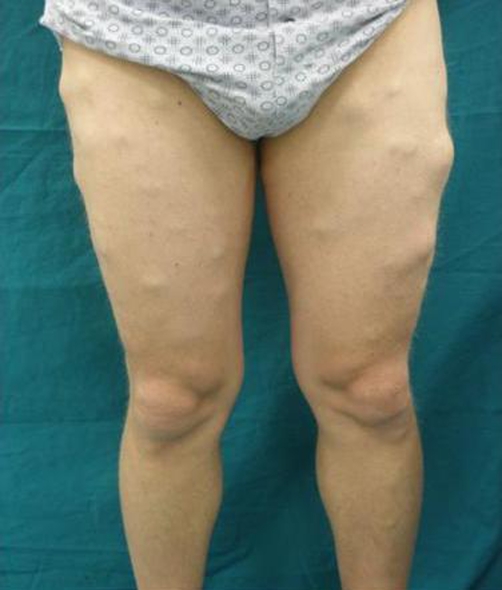
Localization of the three forms of multiple symmetric lipomatosis.

**Figure 2.**
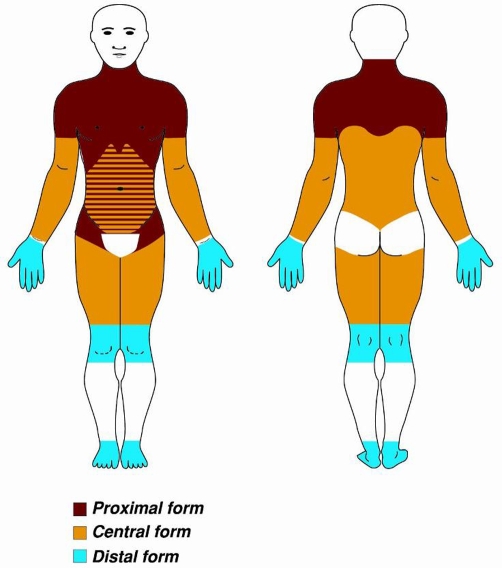
Symmetric, well-circumscribed, grossly round and protruding masses involving the upper legs (anterior aspect).

Clinical examination revealed bilateral, symmetric, soft, well-circumscribed round subcutaneous masses involving the lower trunk, arms and upper legs (Figure 1 and Figure 2). The masses varied in size from 1 cm × 1 cm to 4 cm × 4 cm. The remainder of the physical examination did not reveal abnormalities.

The patient underwent surgery in the supine position. Lipomas on the abdomen and those located anteriorly on the limbs were excised under general anaesthesia. Approximately 40 lipomas were excised at the first operation. Specimens were encapsulated tumours which grossly resembled typical lipomas. Histologically, fat tissue appeared normal; smaller adipocytes with spindle cells without malignant changes were also observed. We are planning a second operation for posterior lipomas on the limbs and back.

## Discussion

MSL is an uncommon disease characterised by non-encapsulated symmetrical accumulation of fat around the neck, shoulder and upper trunk. It rarely involves the lower limbs and lower trunk. MSL can be divided into three forms according to localization of lipomatous masses: proximal, central and distal.

In the proximal form of MSL, the neck, shoulder, and upper trunk are usually involved, but the upper legs, deltoid region, abdomen and upper thigh region are occasionally involved (Figure 1). Approximately 200 cases of the proximal form have been reported in the literature. Enzi *et al.* studied 31 cases of MLS and found that the commonest location of lipomatosis masses at baseline was the sub mental area (92.3%), followed by the nucal region (67.7), dorsal and deltoid areas (54.8%), abdomen (45.2%), upper segments of arms (41.9%), mammary region (32.3%), and upper segment of legs (19.4%). In the distal form, bilateral hands, feet or knees are involved. Until now, five cases of the purely distal form [5-9], and one mixed (proximal form and distal form at the same time) case [10], have been reported. We described, for the first time, a purely central form of MSL in: the lower trunk, arms and upper legs were symmetrically involved, but the buttocks were free of MSL (Figure 1).

Although MSL predominantly affects middle-aged men of Mediterranean origin with a history of alcohol abuse, some cases have also been reported in young adults and children [4]. Some MSL patients without a history of alcohol consumption have also been reported [4]. In the present case, the patient was 16 years old at the onset of the disease. He had been living on the Mediterranean coast for a while. The disease is more frequently diagnosed in middle-aged Mediterranean subjects [4], but several cases have been recently been reported in north European [11], and non-European subjects [5,7,9].

The cause of MSL is not known. Association with alcoholism (60% to 90% of patients) and some metabolic disturbances (abnormal glucose tolerance; excessive secretion of insulin; hyperuricemia; dyslipidemia; renal tubular acidosis; alterations in liver enzyme levels; abnormal function of glands (thyroid, adrenal, pituitary); and abnormal testicular function) are common. A causal relationship has not been established, indicating that MSL may be a syndrome with various underlying causes.

Due to limited etiological insights, attempts at treatment are often disappointing. Abstinence from alcohol and treatment of other metabolic problems may prevent progression in mass size, but not their regression. The main treatment is surgical excision of fat or liposuction, but only if the patient complains of aesthetic deformity or, in the case of compressive complications, because the risk of recurrence is high. We preferred open excision rather than liposuction because it offers a safer and more thorough debulking, and is not as traumatic as initially thought. The use of ultrasound-assisted liposuction has also been reported [12]. Lipoma location should be carefully considered before choosing one technique over another. Medical therapy with thyroid extracts, vitamins and salbutamol (for lipolysis stimulation) has not been proved to be effective [13]. Intra-lesional injection of enoxaparin [14] could be an alternative for the treatment of fat masses in MSL, but efficacies have not been consistently demonstrated.

In conclusion, we described a new form of MSL in a young Mediterranean male. This is the first description of a purely central MSL involving the abdomen, back, hands and upper legs symmetrically.

## Abbreviations

ACTH: adrenocorticotropic hormone
DHEA-SO4: dehydroepiandrosterone sulphate
FSH: follicle stimulating hormone
fT3: free-triiodothyronine
fT4: free-tetraiodothyronine
GH: growth hormone
LH: luteinizing hormone
MSL: multiple symmetric lipomatosis
TSH: thyroid-stimulating hormone
